# Global Identification of Myc Target Genes Reveals Its Direct Role in Mitochondrial Biogenesis and Its E-Box Usage In Vivo

**DOI:** 10.1371/journal.pone.0001798

**Published:** 2008-03-12

**Authors:** Jonghwan Kim, Ji-hoon Lee, Vishwanath R. Iyer

**Affiliations:** Section of Molecular Genetics and Microbiology, Center for Systems and Synthetic Biology, Institute for Cellular and Molecular Biology, University of Texas at Austin, Austin, Texas, United States of America; The Babraham Institute, United Kingdom

## Abstract

The Myc oncoprotein is a transcription factor involved in a variety of human cancers. Overexpression of Myc is associated with malignant transformation. In normal cells, Myc is induced by mitotic signals, and in turn, it regulates the expression of downstream target genes. Although diverse roles of Myc have been predicted from many previous studies, detailed functions of Myc targets are still unclear. By combining chromatin immunoprecipitation (ChIP) and promoter microarrays, we identified a total of 1469 Myc direct target genes, the majority of which are novel, in HeLa cells and human primary fibroblasts. We observed dramatic changes of Myc occupancy at its target promoters in foreskin fibroblasts in response to serum stimulation. Among the targets of Myc, 107 were nuclear encoded genes involved in mitochondrial biogenesis. Genes with important roles in mitochondrial replication and biogenesis, such as POLG, POLG2, and NRF1 were identified as direct targets of Myc, confirming a direct role for Myc in regulating mitochondrial biogenesis. Analysis of target promoter sequences revealed a strong preference for Myc occupancy at promoters containing one of several described consensus sequences, CACGTG, in vivo. This study thus sheds light on the transcriptional regulatory networks mediated by Myc in vivo.

## Introduction

The Myc oncogene is a transcription factor that has important roles in regulation of the cell cycle, proliferation, differentiation, apoptosis, and miRNA activation [1], [2], [3], [4]. Deregulation of Myc expression by translocation causes Burkitt's lymphoma [5], [6]. Several studies have shown that Myc expression is increased in many different tumors, indicating that a high level of Myc favors uncontrolled cell proliferation, inhibits cell differentiation and contributes to tumorigenesis [7].

Several Myc-related genes, such as c-Myc, N-Myc, L-Myc, and S-Myc are known in mammals [8], and among them, c-Myc, N-Myc, and L-Myc have been implicated in the genesis of human tumors [9]. A basic helix-loop-helix leucine zipper (bHLH/LZ) protein, Myc dimerizes with another bHLH/LZ protein, Max, to function as a sequence specific DNA-binding transcription factor 10, 11. Max forms Max-Max homodimers or heterodimerizes with Mad family proteins [9]. Myc-Max heterodimers are believed to generally function as an activator complex and Mad-Max heterodimers function as a repressor complex [12], [13]. However, some studies have shown that Max also mediates Myc-dependent transcriptional repression [14], and that Myc represses the transcription of downstream target genes by interfering with the function of other transcriptional activators such as Miz-1 [15], NF-Y [16], or Sp1 [17]. Given the functions of Myc in various biological processes, identifying the downstream targets of Myc has been an area of active research. Genome-wide analysis using microarrays and serial analysis of gene expression (SAGE) suggest that Myc could regulate several hundred genes in mammalian cells [18], [19]. More than a thousand targets of Myc in mammalian cells have been identified and compiled in public databases, which however include direct as well as secondary targets [20].

More recently, genome-wide chromatin immunoprecipitation studies [14], [21], [22] have been performed in different cellular contexts to identify approximately 1300 direct binding target sites of Myc. These studies suggest that about 15% of all annotated genes are direct targets of Myc. Although some of these studies were comprehensive, the overlap between Myc targets identified in different large scale studies is small. It is thus likely that our knowledge of the targets and therefore, the functions, of this oncogene is still limited, given the possibility that it regulates distinct sets of targets in different cell types and physiological growth conditions. Changes in the binding of Myc to its target sites in different physiological conditions in the same cell type have not been systematically examined. Although many previous studies have implicated Myc regulation of many cellular processes, in general, it is not clear whether regulation by Myc occurs by virtue of its position at the head of a transcriptional regulatory cascade, or whether these processes are regulated directly by Myc binding to the promoters of the relevant targets. Myc is known to bind to a canonical consensus DNA sequence CACGTG, termed the E-box, but has also been reported to bind in vitro to several other non-canonical DNA motifs, such as CATGTG, CATGCG, CACGCG, CACGAG, and CAACGTG [23]. However, it is not clear whether Myc exhibits significant selectivity with regard to these sequence motifs in binding to its target sites in the genome.

Here, we identified 1469 Myc direct binding target genes in HeLa cells and human foreskin fibroblasts using human core promoter microarrays. A majority of these targets have not been identified as Myc targets before and hence are novel. We observed a dramatic increase in Myc binding to its target promoters in response to serum stimulation in normal fibroblasts. A strikingly significant fraction of Myc direct target genes were those involved in mitochondrial biogenesis. Additionally, we also analyzed the usage of Myc consensus sequences in vivo and show that the canonical E-box CACGTG and non-canonical CACGCG sequences are the major motifs that are utilized by Myc in binding its target promoters in vivo.

## Results and Discussion

### Myc binds to many target gene promoters in HeLa cells

**Figure 1:**
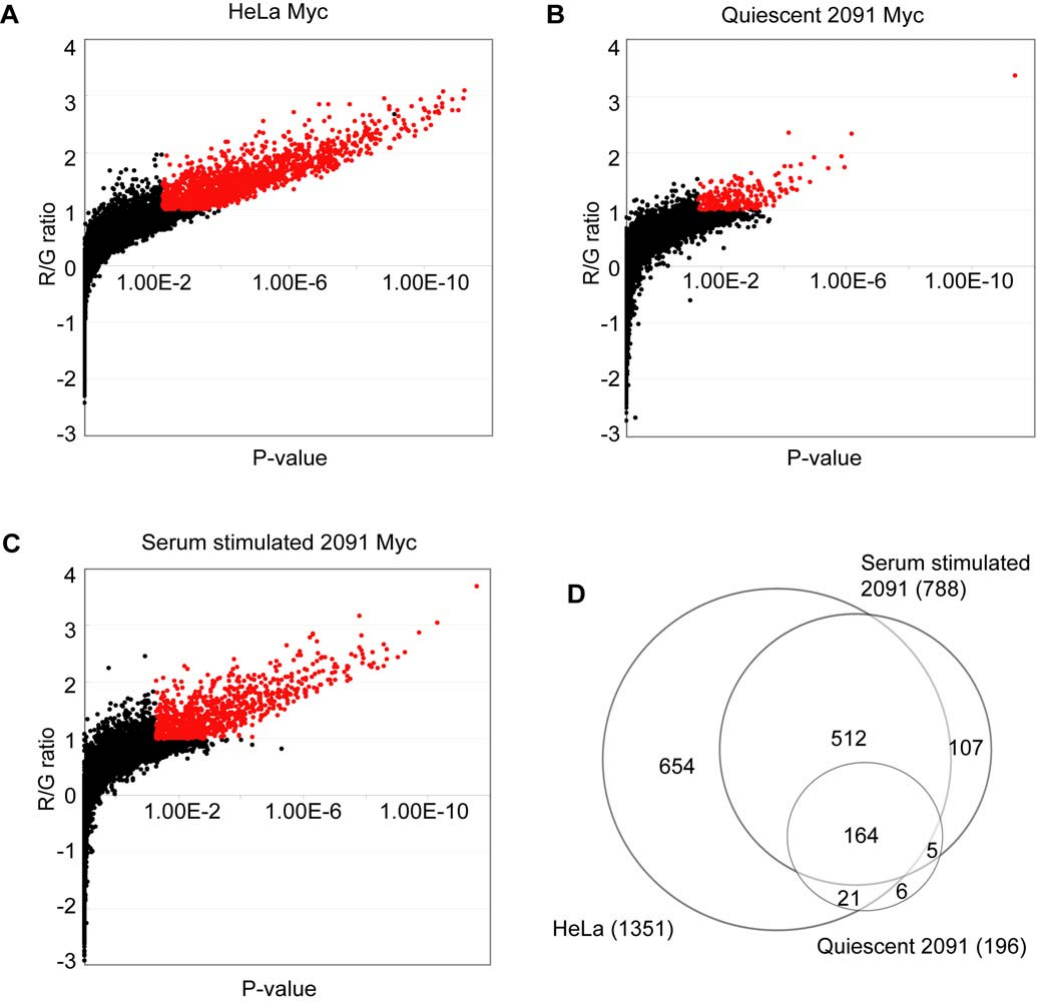
Direct binding targets of Myc in HeLa cells, quiescent foreskin fibroblasts, and serum stimulated foreskin fibroblasts. Fibroblasts are designated “2091”. Target binding loci of Myc were detected by ChIP-chip analysis in (A) HeLa cells, (B) quiescent (serum starved for 3 days) foreskin fibroblasts, and (C) serum stimulated foreskin fibroblasts (for 4 hours). Each scatter plot shows the red/green ratio (log_2_ transformed) on the Y-axis, and the *p*-value in X-axis. Red data points indicate Myc target loci with red/green log_2_ ratio >1 (2-fold), and *p*<0.005. 1351, 196, and 788 promoters were occupied by Myc respectively in the three conditions. (D) The Venn diagram compares target genes in HeLa cells, quiescent foreskin fibroblasts, and serum stimulated foreskin fibroblasts. A total of 1469 target promoters were identified as targets of Myc. Microarray data are also available through NCBI's Gene Expression Omnibus (GEO, http://www.ncbi.nlm.nih.gov/geo/) under GEO Series accession number GSE5197.

We used human promoter microarrays that contained 9,303 proximal promoters to identify the direct targets of Myc in two different human cell lines, HeLa cells and primary foreskin fibroblasts. The genomic regions between –750 bp and +250 bp relative to transcriptional start sites (TSS) of well annotated genes were amplified and printed on the microarrays [24]. We carried out independent ChIP reactions from four independently grown HeLa cell cultures and co-hybridized the ChIP samples together with a mock immunoprecipitated sample as the reference. We determined the median ratio of each spot on the microarray from the four independent hybridizations, and also calculated a *p*-value for significant ratios using a previously described error model [25], [26]. We considered spots with a ratio ≥2.0 and P value ≤0.005 to represent direct Myc target loci. Among the 9,303 promoter spots analyzed, 1351 spots were in this category (Figure 1A). Control hybridizations with differentially labeled mock immunoprecipitated samples did not yield any significantly enriched loci at this threshold (data not shown).

### Occupancy of target promoters by Myc changes rapidly in response to serum activation of quiescent fibroblasts

The response to serum stimulation has been used as a model for studying mammalian cell cycle activation and proliferation [27], [28]. These studies have led to the identification of Myc as a critical downstream element of proliferation signaling cascades. As an immediate early response transcription factor, the expression levels of Myc mRNA and protein are rapidly induced by a variety of growth factors and serum stimulation [29], [30]. Based on the fact that expression of Myc is strongly and rapidly induced by serum stimulation, and the fact that Myc is known to be an important regulator of cell proliferation, we hypothesized that Myc might show changes in binding to its target promoters in response to serum stimulation. To determine if this was the case, we measured the occupancy of Myc at target promoters in quiescent foreskin fibroblasts as well as in fibroblasts after serum stimulation. Two independent cultures of quiescent foreskin fibroblasts grown in low serum (0.1% FBS) media for 72 hours, as well as fibroblasts stimulated by serum (10% FBS) for 4 hours were used for ChIP, and hybridizations were carried out as described for HeLa cells above, using a mock immunoprecipitated sample as reference. We averaged the ratios from duplicate microarray hybridizations and used the same threshold as for HeLa cells to define the target promoters occupied by Myc in foreskin fibroblasts. We detected 196 promoters occupied by Myc in quiescent fibroblasts (Figure 1B), whereas 788 promoters were occupied by Myc in response to serum stimulation (Figure 1C). Our result thus reveals differential binding of Myc to its target promoters under different growth conditions in the same cell type. Interestingly, Myc occupied a much larger number of target promoters in HeLa cells although the HeLa cells used in our study were not synchronized. Altogether, we identified 1469 direct downstream targets of Myc from at least one of the conditions we tested.

**Figure 2:**
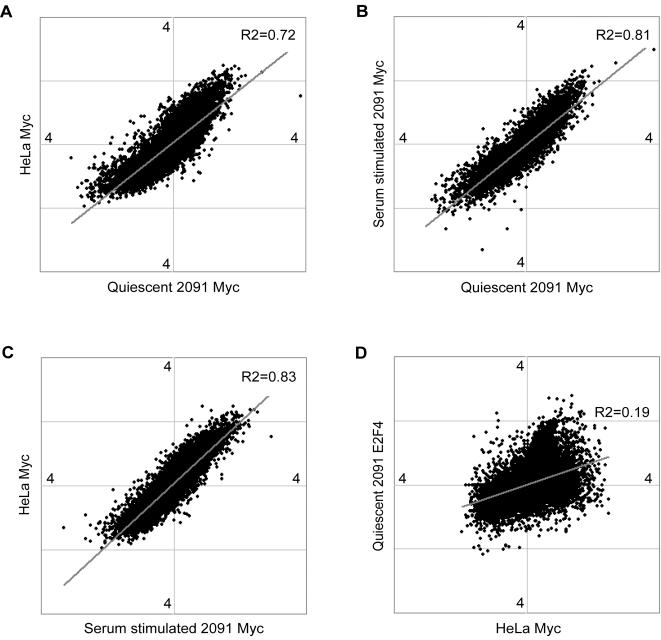
Myc binds largely to the same target loci among different cell types or physiological conditions. Myc binding to promoters is represented by spots in each scatter plot on a log_2_ transformed scale. Comparisons of Myc occupancy at promoters (A) between HeLa cells and quiescent foreskin fibroblasts, (B) between serum stimulated and quiescent foreskin fibroblasts, and (C) between HeLa cells and in serum stimulated foreskin fibroblasts, show a relatively high degree of similarity of Myc binding targets in different cell lines or conditions.(D) As a negative control, a comparison between E2F4 binding to promoters in quiescent fibroblasts and Myc binding to promoters in the same condition was performed. There is little correlation between Myc occupancy and E2F4 occupancy at the promoters (R^2^ = 0.19).

We compared the Myc targets identified in HeLa cells with the Myc targets in fibroblasts to see if there were any differences in target profiles among cell lines or different growth conditions. There were many common Myc targets among the different conditions we examined. Approximately 86% (676 out of 788) of the Myc targets in serum-stimulated fibroblasts were also targets in HeLa cells (Figure 1D). 86% (169 out of 196) of Myc targets in quiescent fibroblasts were also targets of Myc in serum stimulated fibroblasts, and 95% (185 out of 196) of them were also Myc targets in HeLa cells (Figure 1D). Interestingly, a more continuous analysis of target overlaps based on pair-wise global comparisons of each data set (Figure 2A–C) suggested that the occupancy of target promoters by Myc was largely consistent across cell lines and growth conditions. Thus, the differences between its binding targets in HeLa cells and fibroblasts are likely to reflect lower levels of Myc in fibroblasts, rather than its occupancy of a markedly different set of targets in the different cells or conditions.

Since Myc expression is induced by serum stimulation, the small set of genes (27) whose promoters were occupied by Myc in serum starved fibroblasts and not in serum stimulated fibroblasts is noteworthy. We examined expression changes of this subset of genes upon serum stimulation, using an expression data set published from our lab [31]. We found that this subset of Myc targets included genes that are activated and repressed by serum stimulation, as well as genes that show no significant expression change (Supplementary Figure S1). This behavior is consistent with the previously reported role for Myc as a repressor [14], [15], [16], [17], as well as the finding that Myc binding can occur without a corresponding change in mRNA expression levels [22].

A comparison with Myc target genes identified in previously published large-scale experiments [14], [22], [32], [33] also confirmed that there were many targets in common between our study and previous studies, even though each of these studies used different cell lines and different experimental platforms (see Supplementary Table S1). These overlaps do not however reflect some kind of broad ChIP-chip artifact. As a negative control for our pair-wise comparisons, we used data from an E2F4 ChIP-chip experiment. As expected, the Myc occupancy and E2F4 occupancy datasets showed much weaker correlations than the different Myc datasets (Figure 2D).

The 1469 target genes of Myc in HeLa cells and foreskin fibroblasts we report here form one of the largest sets of direct Myc targets identified in a single experiment. 1104 of our 1469 targets were not identified in other recent large-scale experiments [14], [22], [Bibr pone.0001798-Fernandez1] [34] (also listed in Supplementary Table S1). This is likely in part because many of these studies used different cell types. For example, Li *et al* used a smaller promoter microarray to identify direct Myc targets in Burkitt lymphoma cells [33]. However, even though the overlap between the two target sets was modest (46%), it was statistically highly significant (*p*<10^−50^). Based on our results, and extrapolating from the coverage of promoters on this array, we estimate that the number of direct target genes of Myc in human cells totals approximately 16% of all human genes.

### Functional categories of Myc targets

The direct Myc target genes we identified fell into a variety of functional categories that suggested a role for Myc in diverse cellular processes. We used DAVID [35] and FatiGO [36] to quantitate the enrichment of different functional groups of genes among its targets and confirm the involvement of Myc in many processes identified in previous large scale experiments, such as DNA, RNA, and protein metabolism, transport, apoptosis, cell cycle, signal transduction, and cellular proliferation (Supplementary Table S2). KEGG pathway analysis [35] revealed that the Myc direct target genes we identified by ChIP-chip are additionally involved in many previously known and unknown pathways (Table 1). Ribosomal genes comprised one of the most prominent previously known sets of genes that Myc regulates directly, but our analysis identified that it also directly regulates genes involved in oxidative phosphorylation, aminoacyl-tRNA biosynthesis, and glutamate metabolism.

**Table 1:** Functional categories of Myc target genes as indicated by KEGG pathways.

KEGG_PATHWAY: Term	Count	%	PValue
HSA04110: Cell Cycle	25	1.73%	9.67E-05
HSA03010: Ribosome	40	2.77%	1.18E-04
HSA00190: Oxidative Phosphorylation	27	1.87%	5.53E-04
HSA00251: Glutamate Metabolism	10	0.69%	1.28E-03
HSA03030: DNA Polymerase	9	0.62%	1.49E-03
HSA00230: Purine Metabolism	27	1.87%	4.02E-03
HSA00970: Aminoacyl-tRNA Synthetases	9	0.62%	8.07E-03
HSA03060: Protein Export	6	0.42%	9.85E-03
HSA00351: 1,1,1-Trichloro-2,2-Bis(4-Chlorophenyl)Ethane (DDT) Degradation	3	0.21%	2.50E-02
HSA00630: Glyoxylate And Dicarboxylate Metabolism	5	0.35%	2.80E-02
HSA00440: Aminophosphonate Metabolism	6	0.42%	4.17E-02
HSA00350: Tyrosine Metabolism	11	0.76%	5.19E-02
HSA00626: Nitrobenzene Degradation	5	0.35%	5.70E-02
HSA00240: Pyrimidine Metabolism	15	1.04%	6.92E-02
HSA00193: ATP Synthesis	9	0.62%	7.75E-02
HSA00400: Phenylalanine, Tyrosine And Tryptophan Biosynthesis	4	0.28%	7.76E-02

1440 of the 1469 Myc targets were analyzed using the ‘Functional Annotation Tool’ in DAVID (http://david.abcc.ncifcrf.gov/).

### Direct roles of Myc in mitochondrial biogenesis

It is known that Myc activates nuclear-encoded mitochondrial gene expression [18], and that many direct targets of Myc are involved in mitochondrial function [14], [32], [33]. Myc directly regulates TFAM, a transcription factor important for mitochondrial transcription and mitochondrial DNA replication [21], as well as PGC-1β, a regulator of mitochondrial biogenesis [37]. However, the full extent of the relationship between the direct role of Myc acting at its target promoters and mitochondrial function has not been systematically addressed. In addition to these previously reported Myc targets that are involved in mitochondrial biogenesis, it is possible that additional targets in this process are also directly regulated by Myc by its binding to their promoters, indicative of a concerted and multi-pronged regulation of mitochondrial biogenesis by Myc. Indeed, our data and analysis revealed that many functions and pathways that Myc target genes are involved in are closely related to general mitochondrial functions, such as metabolism, apoptosis, oxidative phosphorylation, and glutamate metabolism. It has been suggested that the proliferation and cell cycle progression caused by Myc is in part due to its activation of mitochondrial biogenesis and oxidative phosphorylation [21], [38], [39], which are critical for cell proliferation. However, only a few genes in these pathways have been identified as direct targets of Myc [39]. We found that Myc occupies the promoters of genes such as POLG, POLG2, and NRF1, which have important roles in mitochondrial biogenesis. The promoter of TFBM1, a mitochondrial transcription specificity factor, showed a positive red/green ratio on the promoter microarray, indicative of occupancy by Myc, although it did not meet the *p*-value threshold for defining targets (R/G >2, *p* = 0.0283). NRF1, one of the direct Myc targets we identified, is known to regulate nuclear encoded mitochondrial genes including cytochrome c [39], [40]. Our result thus suggests possible co-regulation of mitochondrial biogenesis by a feed-forward network loop involving Myc and NRF1. It has been previously shown that the apoptotic function of Myc is mediated by NRF1 target genes, and that the balance of mitochondrial genes regulated by Myc and NRF1 is important in cell viability [39]. Our finding suggests that Myc could be an upstream regulator of NRF1 and both of these transcription factors are thus important regulators of mitochondrial biogenesis and diverse mitochondrial functions.

**Figure 3:**
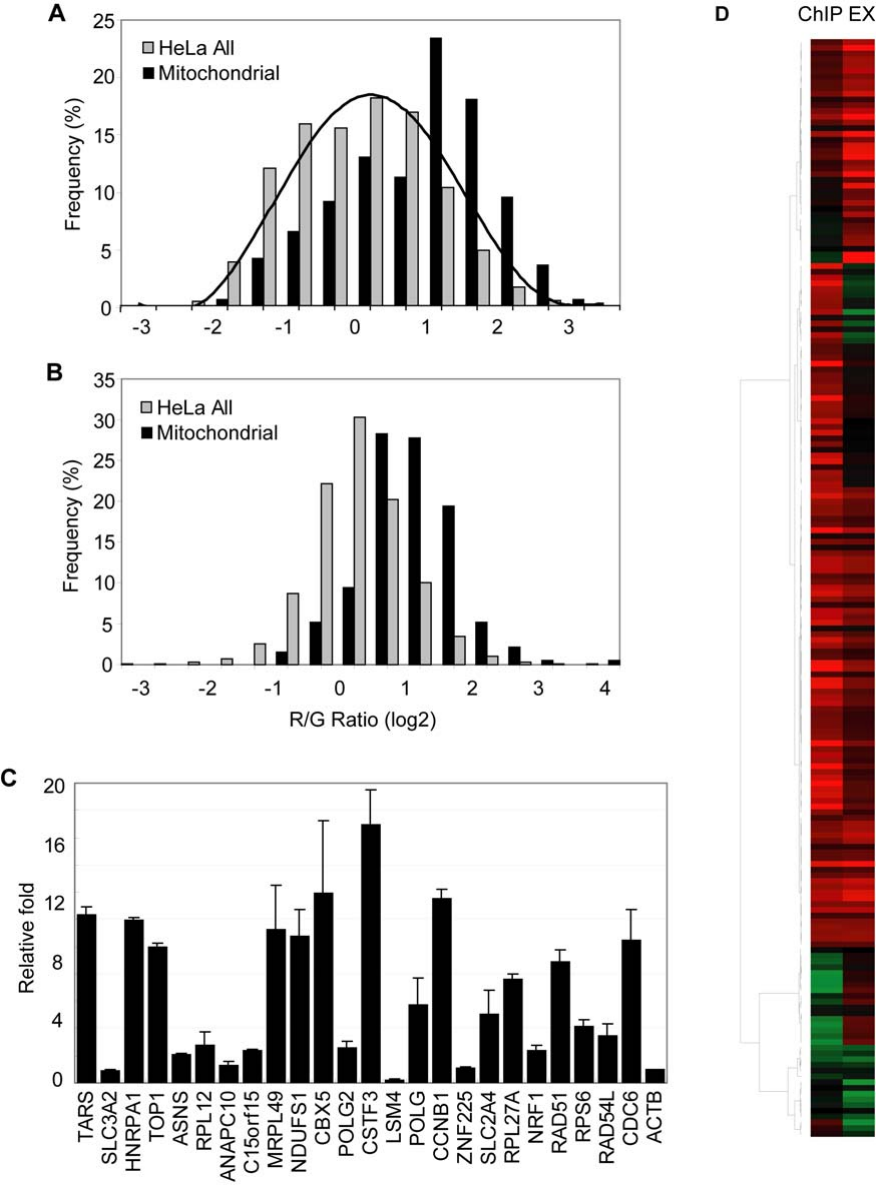
Myc directly regulates genes involved in mitochondrial biogenesis. (A) The occupancy of Myc at all promoters in HeLa cells (gray bars) and at the subset of promoters of genes involved in mitochondrial biogenesis (black bars) is shown. (B) The histogram shows expression profiles of all genes in HeLa cells detected by cDNA microarray (gray bars) and expression of genes involved in mitochondrial biogenesis (black bars). (C) Myc occupancy at the promoters of selected target genes were independently verified by quantitative real-time PCR. The promoter of the actin gene (ACTB) served as a negative control. The graph shows fold enrichment over background, as an average of triplicate PCR reactions from two independent biological replicates. 20 of 24 tested promoters showed significant enrichment compared to the negative control. (D) A gene-by-gene visualization of the correlation between Myc occupancy at the promoters of genes in mitochondrial biogenesis and gene expression in HeLa cells.

In order to systematically quantitate the extent of the involvement of Myc in mitochondrial biogenesis, we searched MitoP2, an integrated database of mitochondrial proteins [41] which lists 785 RefSeq annotated genes, of which promoters for 337 were represented on our promoter arrays. 107 of these 337 mitochondrial genes showed a red/green ratio greater than 2 and are listed in Supplementary Table S3. The red/green ratios of the set of all mitochondrial gene promoters tended to be higher than the overall red/green ratio of all promoters on the array, reflecting higher occupancy of the former set of promoters by Myc (Figure 3A).

The relationship between Myc binding and target gene expression is not a simple one [42]. However, ectopic expression of Myc is known to transcriptionally activate many nuclear genes involved in mitochondrial biogenesis [21]. To determine if this was also the case for the binding targets we identified by ChIP-chip, we examined the level of mitochondrial gene expression in microarray data. In HeLa cells containing high levels of Myc, the expression levels of mitochondrial genes tended to be higher than the background (Figure 3B), which is similar to the pattern of Myc occupancy of its the target promoters (Figure 3A). We independently verified the occupancy by Myc of the promoters of many of these mitochondrial function genes, as well as several other targets identified by our core promoters using quantitative real-time PCR (Figure 3C). A gene-by-gene comparison of Myc binding and target gene expression levels also showed that most of the nuclear mitochondrial genes whose promoters were occupied by Myc were transcriptionally active (Figure 3D). Taken together, our data strongly support the notion that Myc directly binds to the promoters of many nuclear encoded mitochondrial genes and positively regulates the transcription of these genes.

### In vivo usage of E-box elements by Myc

Myc regulates its downstream target genes by binding to cis-acting DNA motifs called E-boxes. The canonical E-box sequences CACGTG and CATGTG [23] as well as non-canonical E-boxes, such as CATGCG, CACGCG, CACGAG, and CAACGTG have been identified as cis-acting elements bound by Myc [43]. Although several E-boxes and E-box derivatives have been identified, the E-box sequence preference of Myc for binding to its chromosomal sites in vivo remains largely unknown. To elucidate the relationship between Myc binding and each of the E-box derivatives occurring in proximal promoters in vivo, we analyzed our ChIP-chip data set.

**Figure 4:**
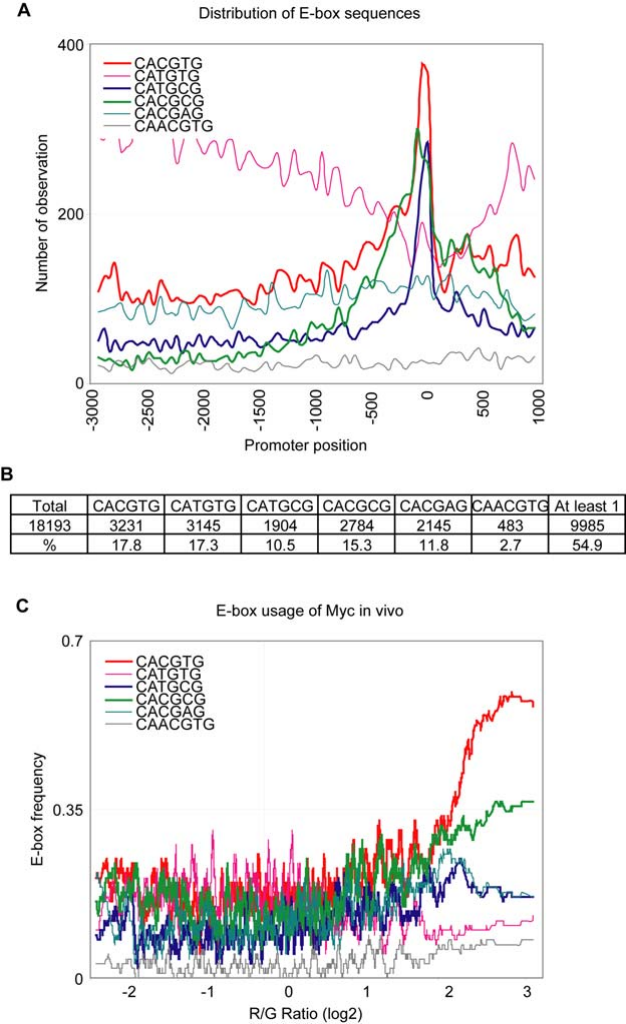
Distribution of E-box elements in human promoters and in vivo usage of E-box sequences in HeLa cells. (A) Distribution (number of occurrences per bin as a function of position relative to the transcription start site (+1, TSS)) of each E-box sequence is shown. Of the six previously defined E-box sequences, CACGTG, CATGCG, and CACGCG were well clustered near the TSS region. A bin size of 50, and a total of 18,193 promoter sequences spanning 3 kb upstream and 1 kb downstream of the TSS were used in this analysis. (B) Number of promoters that contained each E-box element in the region between −800 bp, and +200 bp of the TSS. (C) The relationship between the presence of each E-box sequence in the promoter region (between −800 bp, and +200 bp of the TSS), and Myc binding represented by the log_2_ red/green ratio on the X axis. The Y-axis represents the frequency of each E-box element. A moving window average (size 100) was applied to the frequency of the E-box sequence. The canonical E-box sequence CACGTG, and the non-canonical E-box sequence CACGCG in promoters showed the strongest correlation with Myc binding to its target promoters in HeLa cells.

We first examined the distribution of the previously defined E-box sequences in all human core promoters, considering the regions from 3,000 bp upstream to 1,000 bp downstream of the transcription start sites of 18,193 well-annotated human genes in RefSeq. Three of the six E-box sequences we analyzed–CACGTG, CATGCG and CACGCG–showed a relative enrichment in the promoter region between 800 bp upstream and 200 bp downstream of transcription start site (TSS), whereas in contrast, the sequence CATGTG showed a relative depletion in the same region (Figure 4A). The sequences CACGAG and CAACGTG did not show any significant bias within the 4 kb core promoter region that we analyzed. When we considered only the promoter region between 800 bp upstream and 200 bp downstream, about 55% of all promoters of the RefSeq genes contained at least one of the six E-box elements (Figure 4B). However, each E-box sequence was found in less than 20% of all human promoters.

Approximately 64% of promoters that were occupied by Myc in our ChIP-chip analysis contained at least one of the six E-box sequences. Thus, 36% of the promoters occupied by Myc in vivo lacked any known E-box sequence. We then tested the relationship between the presence of each E-box sequence and Myc occupancy at the promoters. We found that there was a strong correlation between the presence of the canonical E-box sequence CACGTG and Myc occupancy of a promoter. The E-box sequence CACGCG showed lower correlation, while CACGAG, CATGCG, CATGTG, and CAACGTG did not show any significant correlation with Myc binding (Figure 4C). Another postulated binding sequence, CACATG (the reverse complement of CATGTG) also showed no enrichment in Myc target promoters in our data (Supplementary Figure S2), although CACATG/CATGTG have been previously shown to be enriched among Myc binding sites [22]. However, the previous study also examined intronic and intergenic sites which were not included in our promoter arrays, and this could potentially reflect differences in the occurrence of Myc motifs in different types of target sites. Overall, our data show that Myc has a preference to bind only some of the previously defined E-box sequences in vivo. Interestingly although the E-box CATGCG is enriched at proximal promoter regions (Figure 4A) it did not show a significant correlation with Myc occupancy in vivo.

**Figure 5:**
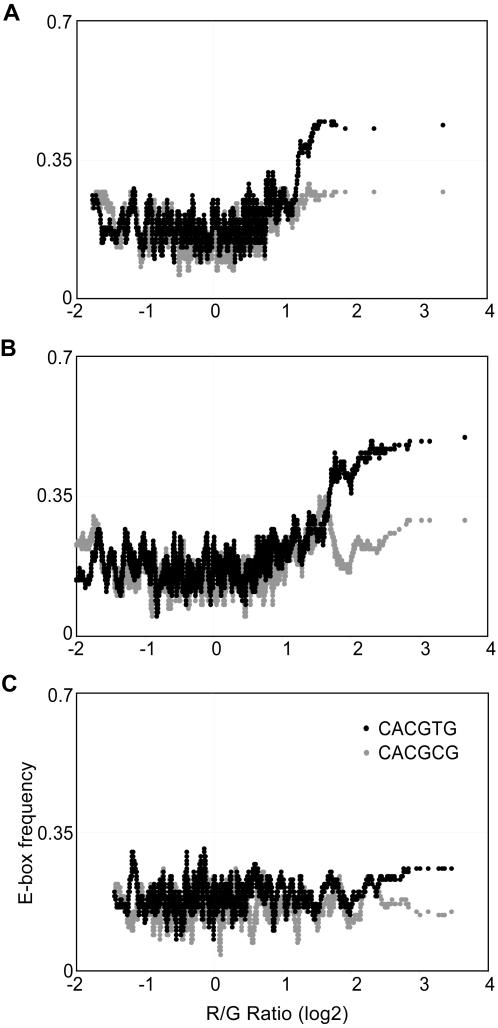
The canonical E-box sequence CATGTG is not preferred by Myc. The relationship between the presence of six E-box sequences in the promoter (between −800 bp and +200 bp of TSS) and Myc binding was analyzed. (A) Top and bottom 2% Myc occupancy data and (B) top and bottom 10% of Myc occupancy data were used. Black bars in both panels represent target promoters that were occupied by Myc and gray bars represent promoters that were not occupied by Myc. Promoters that contained the E-box sequence CATGTG did not show a positive correlation with Myc binding.

To obtain a more detailed understanding of in vivo Myc usage of its binding sites, we examined the relationship between Myc occupancy and the presence of each E-box sequence within the subset of promoters that were most strongly or most weakly occupied by Myc in vivo. More than half the promoters that were most strongly occupied by Myc (the top 2%) contained the CACGTG sequence (Figure 5A). The promoters most strongly occupied by Myc showed a increased frequency of all the previously defined E-box sequences except, interestingly, CATGTG. We observed the same trend when we examined the top or bottom 10% of promoters occupied by Myc (Figure 5B). Our data thus suggest that Myc has a marked preference to utilize promoters in vivo that contain any of the known E-box sequences except CATGTG. However, E-box sequences CACGTG and CACGCG showed the strongest correlations with Myc occupancy (Figure 4C).

**Figure 6:**
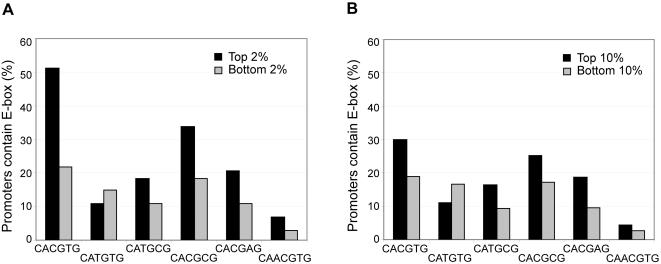
E-box usage in foreskin fibroblasts. The relationship between the presence of the two E-box sequences (CACGTG, and CACGCG) and Myc binding represented by the log_2_ red/green ratio in (A) quiescent foreskin fibroblasts, and (B) in serum stimulated fibroblasts is shown. Only the canonical E-box sequence CACGTG showed good correlation with Myc binding in both conditions. Average moving window (size 100) was applied to the frequency of E-box sequence. (D) E2F4 ChIP-chip data was used as a negative control, and showed no correlation with E-box elements.

To determine whether the most strongly preferred Myc sites, CACGTG and CACGCG, were both also used under distinct physiological conditions that might affect Myc binding, we separately analyzed ChIP-chip data from quiescent and serum-stimulated foreskin fibroblasts. Promoters containing the highly preferred CACGTG sequence were preferentially occupied by Myc in both conditions in foreskin fibroblasts, but promoters with the CACGCG sequence did not show significant correlation with Myc binding (Figure 6A and B). We also did not observe a strong correlation between any of the other E-box sequences and Myc binding in serum-starved fibroblasts, or in serum-stimulated fibroblasts. As a control, we examined the occupancy of E2F4 at these same promoters in quiescent fibroblasts. Despite the fact that there are several common targets for both Myc and E2F4, we did not observe a significant correlation between the presence of the E-box sequences and E2F4 binding, which implies that the E-box sequence is specific to Myc, but not to E2F4 (Figure 6C).

Taken together, our data indicate that the oncoprotein Myc strongly prefers to use promoters containing the E-box sequence CACGTG in vivo, although most of the other E-box sequences except CATGTG appeared to be utilized in vivo to varying extents. The discrepancy between our observations and previous findings might be due to the fact that most of the previous experiments were performed in vitro, and also did not consider the number of actual E-box sequences present in the promoter. We observed an increase in Myc binding to promoters that contain each of the previously defined E-box sequences, except CATGTG. However, the contribution of most E-box sequences to Myc binding in vivo is low except for the E-box sequence CACGTG.

## Materials and Methods

### Cell lines and antibody

Human HeLa cells and human fibroblasts derived from foreskin (ATCC CRL 2091) were grown to about 60% confluency in 15 cm plates in DMEM containing glucose (1 g/liter), antibiotics, and 10% fetal bovine serum (FBS, Hyclone). Exponentially growing HeLa cells were used for ChIP. For the serum starvation experiment, fibroblasts were washed once with phosphate-buffered saline (PBS) and twice with DMEM medium lacking FBS. Low-serum DMEM medium (containing 0.1% FBS) was added to the plates and incubated for 72 hours. For serum activation, cells in low-serum medium were washed twice with PBS, and DMEM medium containing 10% FBS was added to the cells and incubated for 4 hours. For crosslinking, 37% formaldehyde was directly added to the media, and ChIP procedures were subsequently performed using an anti-Myc antibody (sc-764x, Santa Cruz).

### Chromatin immunoprecipitation (ChIP)

HeLa cells and fibroblasts were cross-linked by the addition of formaldehyde (1% final concentration) directly to tissue culture plates and incubated for 7 min at room temperature. Cross-linking was terminated by adding glycine to a final concentration of 125 mM. Cells were washed with cold PBS containing PMSF, scraped off the plates, collected by centrifugation, and washed again with PBS. After centrifugation, the pellet was resuspended in SDS lysis buffer (1% SDS, 10 mM EDTA, 50 mM Tris-Cl pH 8.1, plus protease inhibitors) and incubated at room temperature for 20 min. Cells were sonicated on ice, and fragmented DNA was visualized on an agarose gel (average size 1 kb). The sample was centrifuged at 12000 rpm at 4°C for 10 min, and ChIP dilution buffer (1% Triton X-100, 2 mM EDTA, 20 mM Tris-Cl pH 8.1, 150 mM NaCl, plus protease inhibitors) was added to the collected supernatant. The sample was precleared with protein A-agarose beads (previously washed with ChIP dilution buffer) at 4°C for 1 hour. Precleared chromatin was incubated with an antibody against Myc (sc-764x, Santa Cruz) at 4°C overnight. For the mock IP controls, the antibody was not added. Prewashed protein A-agarose beads were added and protein-DNA complexes were recovered after a 2 hour incubation at 4°C. Immunoprecipitated complexes were successively washed twice with low salt wash buffer (0.1% sodium deoxycholate, 1% Triton X-100, 1 mM EDTA, 50 mM HEPES pH 7.5, 150 mM NaCl), once with high-salt wash buffer (0.1% sodium deoxycholate, 1% Triton X-100, 1 mM EDTA, 50 mM HEPES pH 7.5, 500 mM NaCl), once with LiCl wash buffer (250 mM LiCl, 0.5% NP-40, 0.5% sodium deoxycholate, 1 mM EDTA, 10 mM Tris-Cl pH 8.1), and twice with TE buffer (10 mM Tris-Cl pH 7.5, 1 mM EDTA). SDS elution buffer was added and incubated at 65°C for 30 min to recover protein-DNA complexes. Crosslinks were reversed by incubating at 65 °C overnight. The sample was treated with RNase A and Proteinase K, extracted with phenol/chloroform, and precipitated. The pellet was resuspended in 25 µl of water.

### Ligation mediated PCR (LM-PCR)

LM-PCR was performed as described [26] with minor modifications. LM-PCR consisted of three steps: blunting of ChIP DNA, ligation of linkers to ChIP DNA, and PCR. Approximately 200 ng of immunoprecipitated DNA was diluted to a final volume of 100 µl with water. A mixture of 11 µl 10X T4 DNA polymerase buffer, 0.5 µl BSA (10 mg/ml), 0.5 µl dNTP mix (25 mM each), and 0.2 µl T4 DNA polymerase (3 U/µl) was added, mixed by pipetting, and incubated at 12°C for 20 min. After the incubation, 11.5 µl 3M sodium acetate, and 0.5 µl glycogen (20 mg/ml) were added. The sample was extracted with 120 µl of phenol/chloroform/isoamyl alcohol (25:24:1) in a 0.5 ml Phase-Lock Gel tube (Eppendorf) and centrifuged for 5 min at maximum speed in a microcentrifuge. The upper phase was transferred to a new 1.5-ml Eppendorf tube and precipitated with 230 µl ice-cold ethanol (100%). After centrifugation, the pellet was washed with 70% ethanol and air dried briefly. The pellet was resuspended in 25 µl water and placed on ice. Ligase mix containing 8 µl water, 10 µl 5X ligase buffer, 6.7 µl annealed linkers, and 0.5 µl T4 DNA ligase was added and incubated overnight at 16°C. Linkers were prepared as follows: 300 µl each of oligo Long (40 µM stock, sequence: 5′-GCGGTGACCCGGGAGATCTGAATTC), and oligo Short (40 µM stock, sequence: 5′-GAATTCAGATC) were mixed. 50-µl aliquots of oligo mixture were placed at 95°C for 5 min, 70°C for 5 min and placed at room temperature for 20 min. Aliquots were then transferred to 4°C and allowed to stand overnight. The annealed linkers were stored at −20°C.

To precipitate the linker-ligated DNA, 6 µl of 3 M sodium acetate (pH 5.2) and 130 µl of cold ethanol were added to the tube and centrifuged for 15 min at 4°C. The pellet was washed with 70% ethanol and resuspended in 25 µl water. A mixture of 4 µl 10X PCR buffer, 6.75 µl water, 2 µl low TTP mix (5 mM each dATP, dCTP, dGTP, and 2 mM dTTP), 1 µl Cy3-dUTP or Cy5-dUTP, and 1.25 µl of oligo Long (40 µM), was added to the sample DNA and transferred to PCR tubes. PCR was performed as follows: first, 2 min at 55°C, followed by addition of a mixture of 8 µl water,1 µl 10X PCR buffer, and 1 µl Taq polymerase (5 U/µl ), 5 min at 72°C, 2 min at 95°C, 35 cycles of 30 sec at 95°C, 30 sec at 55°C, and 1 min at 72°C. After PCR amplification, 5 µl of sample was loaded on a 1.5% agarose gel to verify the size of amplified ChIP DNA. The average size of amplified ChIP DNA was approximately 400 bp. The remaining PCR product was purified using a Qiaquick PCR purification kit (Qiagen), and eluted in 13 µl of water.

### Microarrays and data analysis

Human core promoter microarrays were manufactured as described [44]. Amplified and fluorescently labeled ChIP and reference DNA samples were simultaneously hybridized to the microarrays and ChIP enrichment of target loci was calculated based on significant ratios of ChIP signal to reference signal at each spot. Independent biological replicate experiments were combined by averaging the red/green ratio of each spot on microarrays. The confidence of the ratio measurement was calculated as a *p*-value using a previously described error model [25], [26], [45]. Microarray elements whose red/green ratio was ≥2 and *p*-value was ≤0.005 were considered to be direct targets of Myc.

## Supporting Information

Figure S1 Gene expression changes for the subset of genes whose promoters were occupied by Myc in serum starved fibroblasts (designated “2091”) but not in serum stimulated fibroblasts. Expression data is taken from Gu & Iyer (2006) Genome Biol 7: R42, and is shown for the genes in this category for which data was available in the previous dataset.
(0.52 MB TIF)

Figure S2 Occurrence of the CACATG motif among target promoters, compared to the canonical CACGTG and CACGCG motifs. No enrichment is evident.
(0.62 MB TIF)

Table S1 List of 1469 Myc direct target genes identified in this work, and overlap with previously identified targets from global ChIP studies. The occurrence of the consensus motif CACGTG among target promoters is also indicated.
(0.19 MB XLS)

Table S2 Functional categories of Myc target genes identified in this study.
(0.37 MB XLS)

Table S3 Nuclear encoded genes involved in mitochondrial biogenesis whose promoters were occupied by Myc.
(0.03 MB XLS)

## References

[1] Boxer LM, Dang CV (2001). Translocations involving c-myc and c-myc function.. Oncogene.

[2] Grandori C, Cowley SM, James LP, Eisenman RN (2000). The Myc/Max/Mad network and the transcriptional control of cell behavior.. Annu Rev Cell Dev Biol.

[3] O'Donnell KA, Wentzel EA, Zeller KI, Dang CV, Mendell JT (2005). c-Myc-regulated microRNAs modulate E2F1 expression.. Nature.

[4] Pelengaris S, Khan M, Evan G (2002). c-MYC: more than just a matter of life and death.. Nat Rev Cancer.

[5] Dalla-Favera R, Bregni M, Erikson J, Patterson D, Gallo RC (1982). Human c-myc onc gene is located on the region of chromosome 8 that is translocated in Burkitt lymphoma cells.. Proc Natl Acad Sci USA.

[6] Taub R, Kirsch I, Morton C, Lenoir G, Swan D (1982). Translocation of the c-myc gene into the immunoglobulin heavy chain locus in human Burkitt lymphoma and murine plasmacytoma cells.. Proc Natl Acad Sci USA.

[7] Nesbit CE, Tersak JM, Grove LE, Drzal A, Choi H (2000). Genetic dissection of c-myc apoptotic pathways.. Oncogene.

[8] Eisenman RN (2001). Deconstructing myc.. Genes Dev.

[9] Adhikary S, Eilers M (2005). Transcriptional regulation and transformation by Myc proteins.. Nat Rev Mol Cell Biol.

[10] Blackwood EM, Eisenman RN (1991). Max: a helix-loop-helix zipper protein that forms a sequence-specific DNA-binding complex with Myc.. Science.

[11] Blackwood EM, Luscher B, Kretzner L, Eisenman RN (1991). The Myc:Max protein complex and cell growth regulation.. Cold Spring Harb Symp Quant Biol.

[12] Ayer DE, Kretzner L, Eisenman RN (1993). Mad: a heterodimeric partner for Max that antagonizes Myc transcriptional activity.. Cell.

[13] Grandori C, Mac J, Siebelt F, Ayer DE, Eisenman RN (1996). Myc-Max heterodimers activate a DEAD box gene and interact with multiple E box-related sites in vivo.. EMBO J.

[14] Mao DY, Watson JD, Yan PS, Barsyte-Lovejoy D, Khosravi F (2003). Analysis of Myc bound loci identified by CpG island arrays shows that Max is essential for Myc-dependent repression.. Curr Biol.

[15] Herold S, Wanzel M, Beuger V, Frohme C, Beul D (2002). Negative regulation of the mammalian UV response by Myc through association with Miz-1.. Mol Cell.

[16] Izumi H, Molander C, Penn LZ, Ishisaki A, Kohno K (2001). Mechanism for the transcriptional repression by c-Myc on PDGF beta-receptor.. J Cell Sci.

[17] Gartel AL, Ye X, Goufman E, Shianov P, Hay N (2001). Myc represses the p21(WAF1/CIP1) promoter and interacts with Sp1/Sp3.. Proc Natl Acad Sci USA.

[18] Coller HA, Grandori C, Tamayo P, Colbert T, Lander ES (2000). Expression analysis with oligonucleotide microarrays reveals that MYC regulates genes involved in growth, cell cycle, signaling, and adhesion.. Proc Natl Acad Sci USA.

[19] Menssen A, Hermeking H (2002). Characterization of the c-MYC-regulated transcriptome by SAGE: identification and analysis of c-MYC target genes.. Proc Natl Acad Sci USA.

[20] Zeller KI, Jegga AG, Aronow BJ, O'Donnell KA, Dang CV (2003). An integrated database of genes responsive to the Myc oncogenic transcription factor: identification of direct genomic targets.. Genome Biol.

[21] Li F, Wang Y, Zeller KI, Potter JJ, Wonsey DR (2005). Myc stimulates nuclearly encoded mitochondrial genes and mitochondrial biogenesis.. Mol Cell Biol.

[22] Zeller KI, Zhao X, Lee CW, Chiu KP, Yao F (2006). Global mapping of c-Myc binding sites and target gene networks in human B cells.. Proc Natl Acad Sci USA.

[23] Blackwell TK, Huang J, Ma A, Kretzner L, Alt FW (1993). Binding of myc proteins to canonical and noncanonical DNA sequences.. Mol Cell Biol.

[24] Odom DT, Zizlsperger N, Gordon DB, Bell GW, Rinaldi NJ (2004). Control of pancreas and liver gene expression by HNF transcription factors.. Science.

[25] Hu Z, Killion PJ, Iyer VR (2007). Genetic reconstruction of a functional transcriptional regulatory network.. Nat Genet.

[26] Ren B, Robert F, Wyrick JJ, Aparicio O, Jennings EG (2000). Genome-wide location and function of DNA binding proteins.. Science.

[27] Wick M, Burger C, Brusselbach S, Lucibello FC, Muller R (1994). Identification of serum-inducible genes: different patterns of gene regulation during G0–>S and G1–>S progression.. J Cell Sci.

[28] Winkles JA (1998). Serum- and polypeptide growth factor-inducible gene expression in mouse fibroblasts.. Prog Nucleic Acid Res Mol Biol.

[29] Iyer VR, Eisen MB, Ross DT, Schuler G, Moore T (1999). The transcriptional program in the response of human fibroblasts to serum.. Science.

[30] Oster SK, Ho CS, Soucie EL, Penn LZ (2002). The myc oncogene: MarvelouslY Complex.. Adv Cancer Res.

[31] Gu J, Iyer VR (2006). PI3K signaling and miRNA expression during the response of quiescent human fibroblasts to distinct proliferative stimuli.. Genome Biol.

[pone.0001798-Fernandez1] Fernandez PC, Frank SR, Wang L, Schroeder M, Liu S (2003). Genomic targets of the human c-Myc protein.. Genes Dev.

[33] Li Z, Van Calcar S, Qu C, Cavenee WK, Zhang MQ (2003). A global transcriptional regulatory role for c-Myc in Burkitt's lymphoma cells.. Proc Natl Acad Sci USA.

[34] Bolton EC, Boeke JD (2003). Transcriptional interactions between yeast tRNA genes, flanking genes and Ty elements: a genomic point of view.. Genome Res.

[35] Dennis G, Sherman BT, Hosack DA, Yang J, Gao W (2003). DAVID: Database for Annotation, Visualization, and Integrated Discovery.. Genome Biol.

[36] Al-Shahrour F, Diaz-Uriarte R, Dopazo J (2004). FatiGO: a web tool for finding significant associations of Gene Ontology terms with groups of genes.. Bioinformatics.

[37] Zhang H, Gao P, Fukuda R, Kumar G, Krishnamachary B (2007). HIF-1 inhibits mitochondrial biogenesis and cellular respiration in VHL-deficient renal cell carcinoma by repression of C-MYC activity.. Cancer Cell.

[38] Morrish F, Hockenbery D (2003). Myc's mastery of mitochondrial mischief.. Cell Cycle.

[39] Morrish F, Giedt C, Hockenbery D (2003). c-MYC apoptotic function is mediated by NRF-1 target genes.. Genes Dev.

[40] Virbasius JV, Scarpulla RC (1994). Activation of the human mitochondrial transcription factor A gene by nuclear respiratory factors: a potential regulatory link between nuclear and mitochondrial gene expression in organelle biogenesis.. Proc Natl Acad Sci USA.

[41] Andreoli C, Prokisch H, Hortnagel K, Mueller JC, Munsterkotter M (2004). MitoP2, an integrated database on mitochondrial proteins in yeast and man.. Nucleic Acids Res.

[42] Patel JH, Loboda AP, Showe MK, Showe LC, McMahon SB (2004). Analysis of genomic targets reveals complex functions of MYC.. Nat Rev Cancer.

[43] James L, Eisenman RN (2002). Myc and Mad bHLHZ domains possess identical DNA-binding specificities but only partially overlapping functions in vivo.. Proc Natl Acad Sci USA.

[44] Kim J, Bhinge AA, Morgan XC, Iyer VR (2005). Mapping DNA-protein interactions in large genomes by sequence tag analysis of genomic enrichment.. Nat Methods.

[45] Roberts CJ, Nelson B, Marton MJ, Stoughton R, Meyer MR (2000). Signaling and circuitry of multiple MAPK pathways revealed by a matrix of global gene expression profiles.. Science.

