# Supplementary figures and images for: Global Identification of Myc Target Genes Reveals Its Direct Role in Mitochondrial Biogenesis and Its E-Box Usage In Vivo
Source: PLoS One. 2008 Mar 12;3(3):e1798. doi: 10.1371/journal.pone.0001798 (PMC2258436; doi:10.1371/journal.pone.0001798)

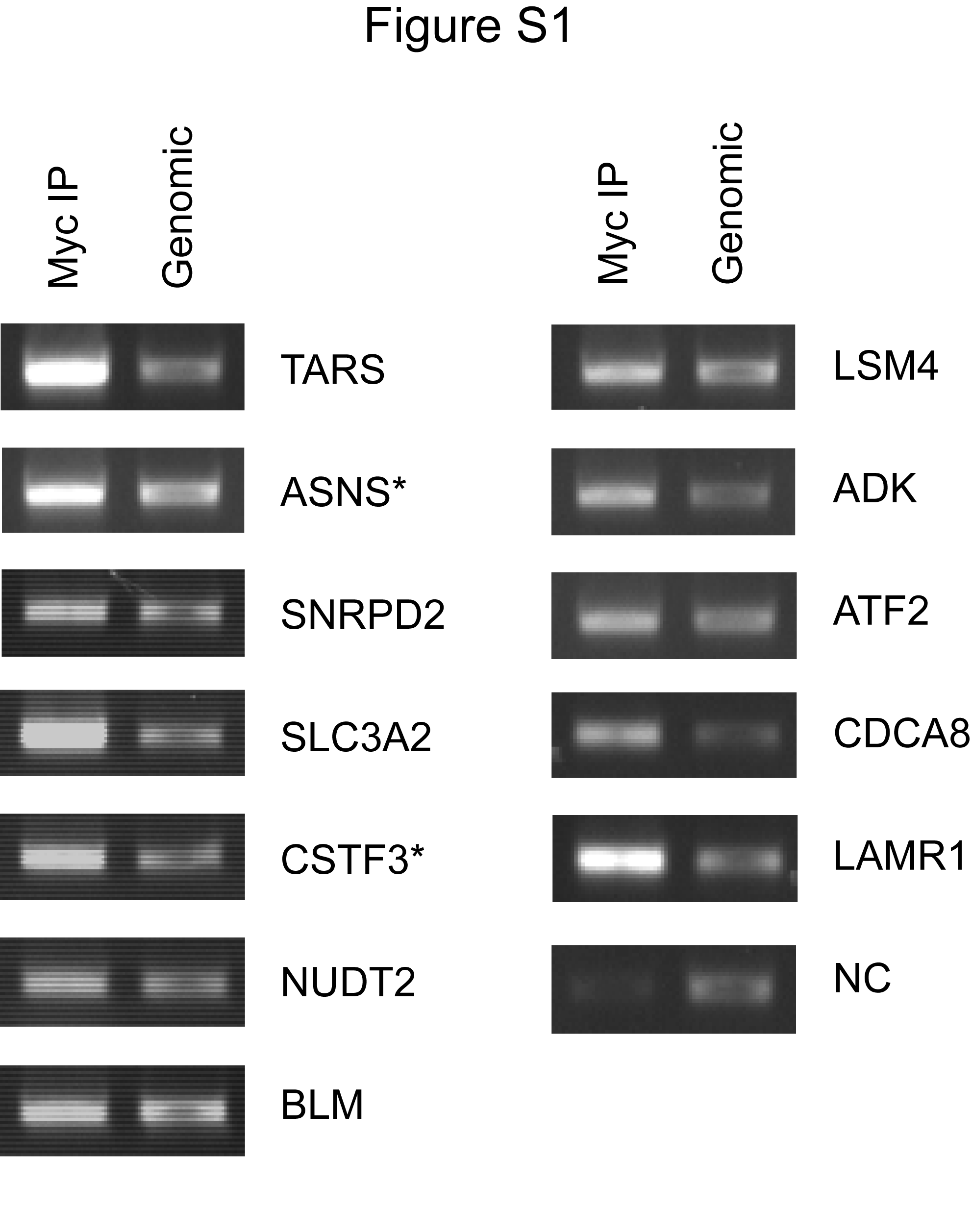

Supplement: Figure S1 — Gene expression changes for the subset of genes whose promoters were occupied by Myc in serum starved fibroblasts (designated “2091”) but not in serum stimulated fibroblasts. Expression data is taken from Gu & Iyer (2006) Genome Biol 7: R42, and is shown for the genes in this category for which data was available in the previous dataset. (0.52 MB TIF) [file pone.0001798.s001.tif]

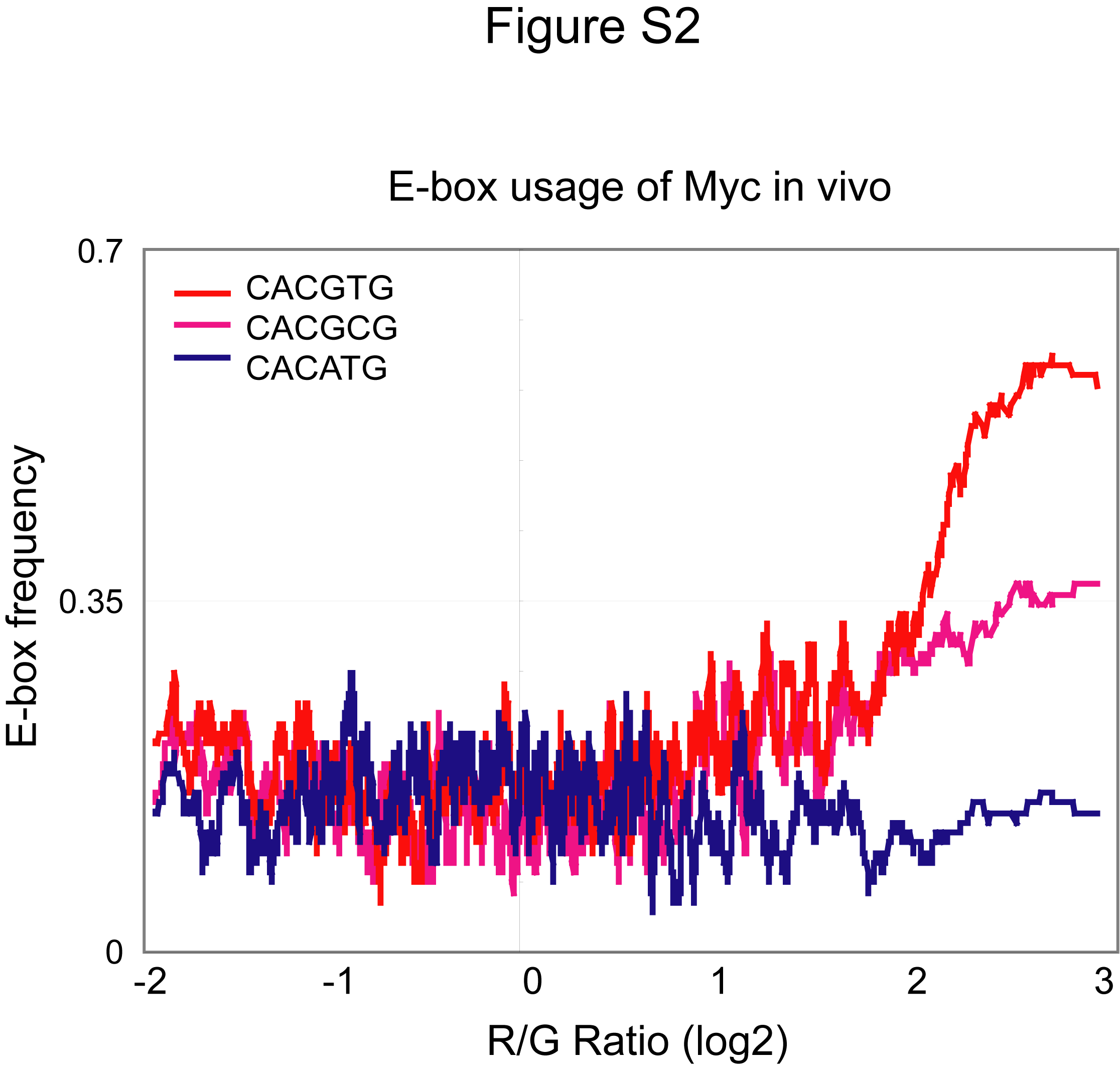

Supplement: Figure S2 — Occurrence of the CACATG motif among target promoters, compared to the canonical CACGTG and CACGCG motifs. No enrichment is evident. (0.62 MB TIF) [file pone.0001798.s002.tif]
